# Acute decompensated left heart failure in a young patient revealing a large obstructive left ventricular mass

**DOI:** 10.11604/pamj.2021.40.79.31056

**Published:** 2021-10-06

**Authors:** Houda Mokhlis, Achraf Zaimi

**Affiliations:** 1 Department of Cardiology, Mohammed V Military Hospital, Mohammed V University, Rabat, Morocco

**Keywords:** Left heart failure, ventricular mass, thrombus

## Image in medicine

We report the case of a 21-year-old female patient with no prior cardiovascular history, at only 3 months postpartum, who was presented to the emergency room for rapidly worsening dyspnea, the physical examination showed low O_2_ stats and crackling lung sounds with signs of respiratory struggle, electrocardiograms (EKG) showed a regular sinus tachycardia at 145bpm with negative T waves in apicolateral leads. The patient was intubated and benefited from an emergency thoracic computed tomography (CT) angiography which showed a bilateral hilar alveolar-interstitial syndrome without signs of pulmonary embolism, the transthoracic echocardiography showed a huge obstructive mass of the left ventricle with segmental kinetic disorders. To better understand the nature of this mass a transoesophageal echocardiography was performed which was in favour of a thrombus, unfortunately, the patient died the same day before any procedure could have been done.

**Figure 1:**
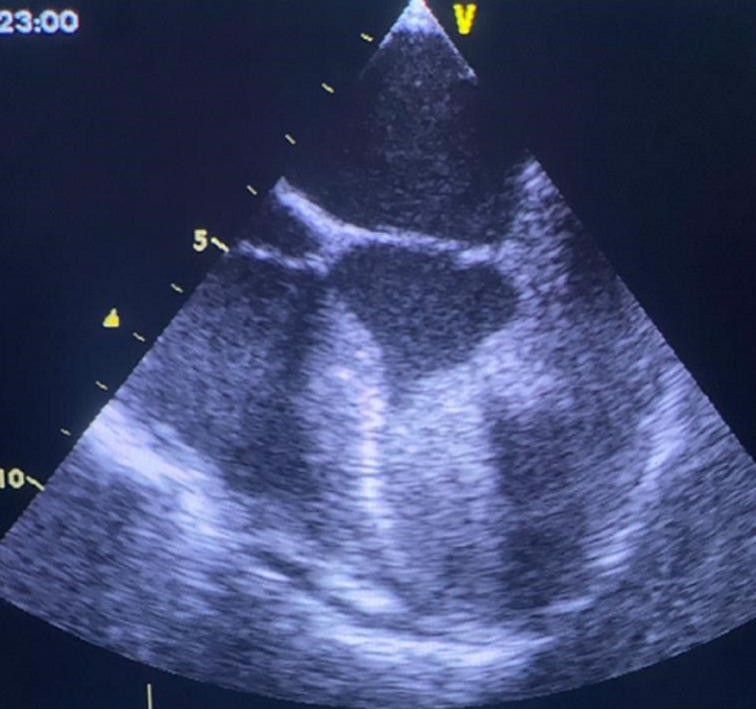
transesophageal echocardiography image showing the mass

